# On timing-optimized SiPMs for Cherenkov detection to boost low cost time-of-flight PET

**DOI:** 10.1088/1361-6560/ace8ee

**Published:** 2023-08-09

**Authors:** Stefan Gundacker, Giacomo Borghi, Simon R Cherry, Alberto Gola, Daehee Lee, Stefano Merzi, Michele Penna, Volkmar Schulz, Sun Il Kwon

**Affiliations:** 1 Department of Physics of Molecular Imaging Systems, Institute for Experimental Molecular Imaging, RWTH Aachen University, Forckenbeckstrae 55, D-52074 Aachen, Germany; 2 Fondazione Bruno Kessler, via Sommarive 18, Trento I-38123, Italy; 3 Department of Biomedical Engineering, University of California, Davis, One Shields Avenue, Davis, CA 95616, United States of America; 4 Dipartimento di Elettronica e Telecomunicazioni, Politecnico di Torino, Corso Duca degli Abruzzi 24, I-10129 Torino, Italy

**Keywords:** fast timing, BGO, FBK NUV-HD, Cherenkov, TOF-PET, single photon time resolution (SPTR), coincidence time resolution (CTR)

## Abstract

*Objective.* Recent SiPM developments and improved front-end electronics have opened new doors in TOF-PET with a focus on prompt photon detection. For instance, the relatively high Cherenkov yield of bismuth-germanate (BGO) upon 511 keV gamma interaction has triggered a lot of interest, especially for its use in total body positron emission tomography (PET) scanners due to the crystal’s relatively low material and production costs. However, the electronic readout and timing optimization of the SiPMs still poses many questions. Lab experiments have shown the prospect of Cherenkov detection, with coincidence time resolutions (CTRs) of 200 ps FWHM achieved with small pixels, but lack system integration due to an unacceptable high power uptake of the used amplifiers. *Approach.* Following recent studies the most practical circuits with lower power uptake (<30 mW) have been implemented and the CTR performance with BGO of newly developed SiPMs from Fondazione Bruno Kessler tested. These novel SiPMs are optimized for highest single photon time resolution (SPTR). *Main results.* We achieved a best CTR FWHM of 123 ps for 2 × 2 × 3 mm^3^ and 243 ps for 3 × 3 × 20 mm^3^ BGO crystals. We further show that with these devices a CTR of 106 ps is possible using commercially available 3 × 3 × 20 mm^3^ LYSO:Ce,Mg crystals. To give an insight in the timing properties of these SiPMs, we measured the SPTR with black coated PbF_2_ of 2 × 2 × 3 mm^3^ size. We confirmed an SPTR of 68 ps FWHM published in literature for standard devices and show that the optimized SiPMs can improve this value to 42 ps. Pushing the SiPM bias and using 1 × 1 mm^2^ area devices we measured an SPTR of 28 ps FWHM. *Significance.* We have shown that advancements in readout electronics and SiPMs can lead to improved CTR with Cherenkov emitting crystals. Enabling time-of-flight with BGO will trigger a high interest for its use in low-cost and total-body PET scanners. Furthermore, owing to the prompt nature of Cherenkov emission, future CTR improvements are conceivable, for which a low-power electronic implementation is indispensable. In an extended discussion we will give a roadmap to best timing with prompt photons.

## 1. Introduction

In positron emission tomography (PET), time-of-flight (TOF) information, which measures the difference in arrival time of the two 511 keV gamma photons, spatially constrains the location of each annihilation event, leading to improved signal-to-noise ratio (SNR) in a PET scan. When a 511 keV gamma ray interacts with a high refractive index media, prompt Cherenkov photons can be produced. These Cherenkov photons are faint but have the potential to improve coincidence timing resolution (CTR) by coupling a Cherenkov radiator to high photon detection efficiency (PDE) SiPMs (Kwon *et al* 2016, Brunner and Schaart 2017, Cates and Levin 2019, Gundacker and Heering 2020) or integrating a Cherenkov radiator into the multichannel plate photomultiplier tube (MCP-PMT) structure (Ota *et al* 2019, 2020, Kwon *et al* 2021). Recently, bismuth germanate (BGO) is getting attention as an emerging scintillator for use in TOF-PET applications, because its CTR was dramatically improved by detecting Cherenkov photons produced in BGO, resulting in a CTR of sub-300 ps when BGO is coupled to SiPMs (Brunner and Schaart 2017, Cates and Levin 2019, Gundacker *et al* 2020). Thus, the timing performance is approaching that of conventional TOF-PET scanners. Because the cost of BGO is estimated to be about three times lower than for commonly used LYSO:Ce, BGO is one of the most promising candidates for low-cost PET detectors, especially in view of large total body PET scanners (Badawi *et al* 2019). The cost effectiveness of BGO is mediated via a lower melting temperature as compared to LYSO, allowing for the use of different low cost crucibles and the lower raw material prices in the case of BGO.

However, only a small number of Cherenkov photons (∼17) are produced in BGO by a 511 keV interaction (Gundacker *et al* 2020), therefore, to obtain a good CTR it is of utmost importance to use SiPMs with very high PDE, excellent single photon time resolution (SPTR) and low correlated noise. Furthermore, the electronic readout is a crucial component in achieving highest time resolution with BGO and to benefit from a high PDE and especially from a superb and improved SPTR. Key aspects in the readout are high electronic bandwidth and low noise, which have to be achieved with a reasonable power consumption. Current work has shown the possibilities of such electronics developments (Cates and Choong 2022, Krake *et al* 2022), which can be seen as a milestone in implementing such concepts in systems and also plays back the ball of action to the SiPM development, i.e. on PDE and SPTR improvements. Additionally, Monte-Carlo simulations have shown the potential of double-sided readout, which could lead to sub-100 ps CTRs with BGO (He *et al* 2023), if the SiPM and electronics are optimized. Furthermore, new readout concepts in prompt gamma imaging for heavy-ion and proton therapy are emerging, also in the need of fast detectors (Jacquet *et al* 2023).

In this study, we developed and tested new SiPM technologies aimed at obtaining optimal timing performance, especially in the domain of highest SPTR, for BGO-based TOF-PET detectors. Experiments were performed independently at multiple sites, Fondazione Bruno Kessler (FBK), University of California, Davis, and RWTH Aachen University, to evaluate the performance by different means and experimental conditions.

## 2. Materials and methods

### 2.1. Novel NUV-HD-CHK SiPMs

**Figure 1.**
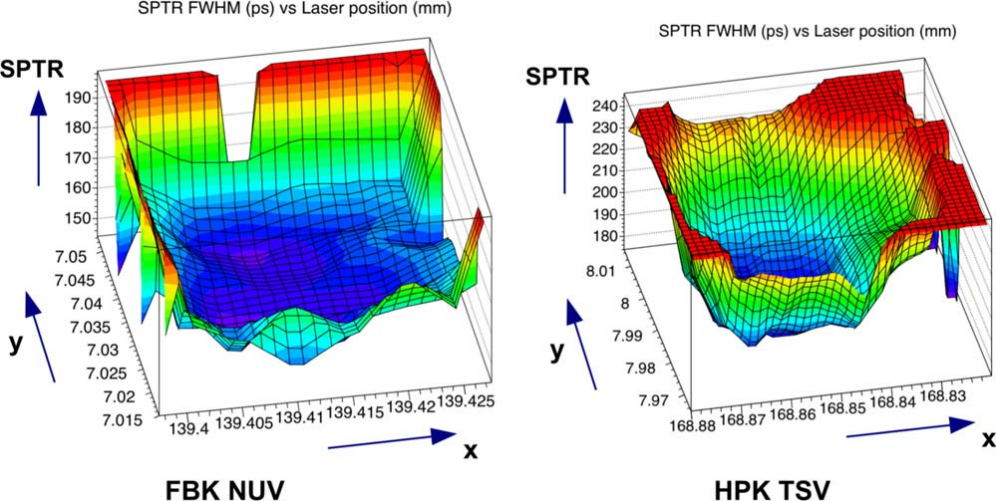
SPTR measured with a focused laser beam (∼1 *μ*m), reveals worse SPTR at the edges of the single photon avalanche diode (Nemallapudi *et al* 2016, Acerbi and Gundacker 2019).

Former focused picosecond-laser measurements have shown that the single photon avalanche (SPAD) edges are to be considered if highest SPTR performances should be achieved (Nemallapudi *et al* 2016). An illustration of focused SPTR measurements within a single SPAD for two different SiPMs are shown in figure 1. The main reason of a worse SPTR at the edges was explained by the transition from zero field at the SPAD boarder to the high field region in the middle of the cell. In figure 1 it can be seen that the transition is relatively sharp for devices from FBK with a breadkdown voltage of about 26V and more gradually for devices from Hamamatsu with a breakdown voltage of about 55 V. It can be concluded that this transition region is also a design parameter, but cannot be avoided completely. A viable way to further improve the SPTR is to shield the relatively small boarder of the SPAD edges. From figure 1 it can be inferred that a metal mask with an overlap of 1–3 *μ*m with the SPAD active area might be able to completely shield this transition region from impinging light.

In order to test this hypothesis and to apply a possibly improved SPTR to BGO and Cherenkov readout, FBK has developed the following technologies to improve SiPM timing performance relative to NUV-HD SiPMs, referred to as NUV-HD-CHK SiPMs, where CHK stands for the Cherenkov optimization.

• Micro-fabrication process: the standard NUV-HD process has been modified to engineer the electric field inside the microcell, in order to have different trade-offs in terms of PDE as a function of the overvoltage (OV), spectral response, electrical characteristics, etc. In the first variation (W15 or low field), PDE increases faster with OV but is lower at longer wavelengths. In the second one (W13 or low field version 2), PDE increases more slowly with OV but is higher at longer wavelengths.
• Micro-cell structure: a metal mask with variable width has been implemented on the micro-cell border of the SiPMs. This solution is expected to improve the SPTR because (i) it produces a higher fast component in the micro-cell signal through an increased capacitive coupling between the anode and the readout pad (increased quenching capacitance *C*_q_), (ii) depending on its width, it can mask the lower-electric-field regions at the edges of the micro-cell, featuring a worse SPTR compared to the central region (Nemallapudi *et al* 2016) and (iii) the metal mask helps to effectively transport the electrical signal to the SiPM anode and, hence, decreases possible signal transfer time delays.

**Figure 2.**
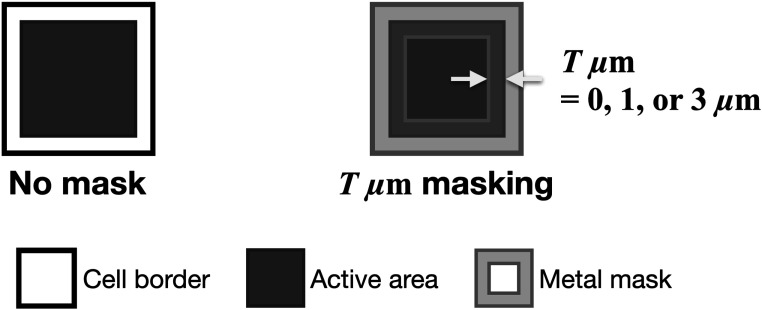
Illustration of the SPADs (cells) with no mask (left) and masking (right) with an overlap of T *μ*m thickness. The SPAD pitch is 40 *μ*m and the SiPMs have an active area of 3 × 3 mm^2^.

In this work, different 3 × 3 mm^2^ NUV-HD-CHK SiPM samples with no mask (NM) and with three different mask configurations showing an overlap of 0 *μ*m, 1 *μ*m and 3 *μ*m (M0, M1 and M3) have been tested, produced in both modified SiPM fabrication processing, i.e. W15—low field (LF) and W13—low field v2 (LF2). An illustration showing the implementation of the metal mask can be seen in figure 2. Regarding the PDE we can calculate, from pure geometric considerations, that M1 and M3 will have a relative PDE loss of 89.4% and 70.3%, respectively. The devices produced are of 3 × 3 mm^2^ size with a SPAD pitch of 40 *μ*m. The breakdown voltage is 32 V. The 3 × 3 mm^2^ SiPMs were evaluated via SPTR and CTR measurements with a coupled PbF_2_, LYSO:Ce,Mg and BGO of size 2 × 2 × 3 mm^3^ and 3 × 3 × 20 mm^3^. Furthermore, SPTR was measured with 1 × 1 mm^2^ sized SiPMs with different masking (NM, M0 and M3) to investigate the impact of the device area.

### 2.2. Experimental setup at FBK

Intrinsic properties of the different NUV-HD-CHK SiPMs were measured at FBK. Besides the *I*–*V* curves, PDE, gain and noise properties were measured. These measurements ensured a proper functionality of the produced samples and characterized the direct and correlated noise variations with the additional implementation of the metal masks.

### 2.3. Experimental setup at UC Davis

**Figure 3.**
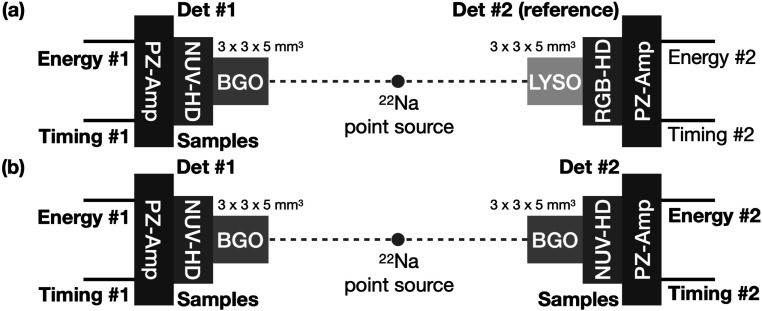
Experimental setup for (a) characterizing different SiPMs and (b) measuring CTR between two selected SiPMs coupled to BGO.

Coincidence events from a ^22^Na point source were acquired using a reference detector and a test detector, as shown in figure 3, in order to characterize SiPM samples fabricated with different technologies. The test detector consisted of a polished 3 × 3 × 5 mm^3^ BGO crystal coupled to each SiPM sample, which was with masking, M0 or M3 from the two implementations W15 (LF) and W13 (LF2).

The BGO pixel was wrapped with Teflon tape and remained unchanged during the characterization studies for all SiPM samples. The reference detector consisted of a polished 3 × 3 × 5 mm^3^ LYSO:Ce coupled to RGB-HD SiPM (FBK, Italy). The RGB-HD SiPM has a broad and high PDE across the visible light region with a maximum value over 50% at 500–550 nm (Kwon *et al* 2016), while the NUV-HD SiPM has its peak PDE at 400–420 nm. Each SiPM was connected to a custom amplifier based on fast operational amplifiers (AD8000) in trans-impedance mode, produced by FBK. The amplifier has two outputs: an energy output used to calculate energy of each event and a timing output filtered with a pole-zero cancellation circuit (Gola *et al* 2013). Output signals were digitized with an oscilloscope (DPO71254C, Tektronix). Coincidence events were determined by a coincidence logic unit using energy signals. All events were acquired biasing SiPMs at ∼6.5 V above breakdown. After characterizing the SiPM samples with a reference detector (figure 3), some SiPMs were selected to measure CTRs between two identical BGO/SiPM detectors, using pairs of SiPMs fabricated with the same design.

### 2.4. Experimental setups at RWTH-Aachen

We measured the CTR with BGO and LYSO:Ce codoped with Mg, whereas the SPTR was measured with black painted PbF_2_ coupled to the NUV-HD-CHK SiPMs, introduced in section 2.1. For the experiments we used a refined high-frequency electronic readout (Gundacker *et al* 2019, Krake *et al* 2022) developed at physics of molecular imaging systems (PMI), RWTH University, Aachen, Germany based on the work of (Cates *et al* 2018).

#### 2.4.1. Power efficient high-frequency (HF) electronics

**Figure 4.**
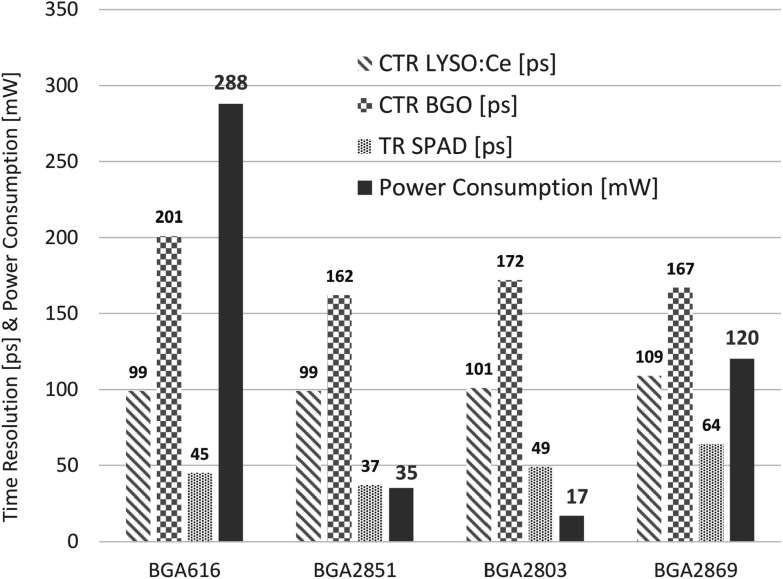
Overview of the CTR achieved with LYSO:Ce (3 × 3 × 3 mm^3^) and BGO (2 × 2 × 3 mm^3^) with the studied amplifiers (Krake *et al* 2022). Along the CTR values the electronic noise contribution to the single SPAD signals (TR_SPAD_) and the power consumption per amplifier are given as well. The BGA616 type was already tested in literature (Gundacker *et al* 2019). The obtained CTR of 99 ps with LYSO:Ce compares well to literature values of 106 ps using similar crystals and SiPMs (Gundacker and Heering 2020). The SiPMs used for the TR_SPAD_ measurements and with LYSO:Ce were from Hamamatsu (HPK) type S14160-3050HS (3 × 3 mm^2^ active area and 50 *μ*m SPAD pitch), whereas BGO measurements were performed with Broadcom SiPMs AFBR-S4N33C013 (3 × 3 mm^2^ active area and 30 *μ*m SPADs).

As already discussed, the detection of prompt photons with analog-SiPMs calls for specialized high-frequency (HF) electronics with bandwidths higher 1 GHz (Gundacker *et al* 2019), which is usually in the need of a high power consumption given by the monolithic microwave integrated circuits (MMICs) used. As an example, components studied in literature (e.g. BGA616) have a current consumption of up to 60mA and a power dissipation of about 300 mW per transistor (Gundacker *et al* 2019). Therefore, the proper electronic readout of SiPMs in the high-frequency domain was carefully revisited and several comparable MMIC amplifiers on the market with low power consumption, high bandwidth, high amplification gain and low noise tested. A complete overview of the used circuit diagram can be found in Krake *et al* (2022). A summary of the results can be seen in figure 4, with the conclusion that all tested amplifiers achieve similar CTR performance with BGO and LYSO:Ce, although having very different power uptake from 288 to 17 mW per single amplifier chip (Krake *et al* 2022). In the following we used the BGA2851 MMIC to measure the SPTR and CTR with the various SiPMs tested.

#### 2.4.2. Coincidence time resolution (CTR)

The CTR was measured in a setup similar as depicted in figure 3 and introduced in Gundacker *et al* (2019). The signals of the SiPMs were read out by ultra-fast HF electronics with power-optimized timing channels as described in section 2.4.1 (see figure 4). Signals were digitized via a Lecroy Waverunner 9404M-MS (bandwidth 4GHz and 40 Gs s^−1^ sampling rate). The timing data was directly digitized on the oscilloscope via a leading edge discrimination, whereas the area under the not amplified SiPM signals was used to calculate and store the energy deposit in the crystal. In the offline data analysis the stored time stamps were selected for energy values around the photopeak in a ±2 sigma environment. It should be noted that no additional baseline correction was performed offline, but rather the front-end design was equipped with a carefully balanced pole-zero baseline fluctuation cancellation circuit.

The detected number of Cherenkov photons underlies a strong fluctuation, which stems from a stochastic photon production related to the path length of the hot recoil electron and the Poisson statistics of light transport and photon detection inefficiencies. In former studies we have demonstrated that the time signal rise time is, to a certain extend, correlated with the number of Cherenkov photons detected (Kratochwil *et al* 2020). In order to account and correct for this fluctuation we measured the timing signal rise time with a two leading edge threshold approach, where the lower threshold was set at 20 mV and the higher threshold at 200 mV. These values have to be set in relation to the single SPAD signal height of about 130 mV for the used SiPMs at an overvoltage of 6 V, which represents the optimum for the CTR measurements. The measured signal rise time was then used to apply a time walk correction on the time stamps as described in Kratochwil *et al* (2020). Furthermore, we used the rise time to classify the events similar to the method used in Kratochwil *et al* (2020).

The CTR spectra is fitted with a sum of two Gaussian, one accounting for fast Cherenkov detection and the other for the slow BGO scintillation, as described in Kratochwil *et al* (2020). On the fit we numerically calculate the full width at half maximum (FWHM), given as the CTR, and the FWTM.

#### 2.4.3. Single photon time resolution

The SPTR was determined using a similar setup as for CTR measurements with one arm modified to hold a small 2 × 2 × 3 mm^3^ PbF_2_ crystal, purchased from EPIC-crystals. PbF_2_ is a sole Cherenkov emitter which produces around 16 prompt photons upon 511 keV gamma absorption (Kratochwil *et al* 2021). In order to reduce optical photon reflections, the crystal surface was painted in black with mat paint of ∼1.4 refractive index. This reduces the photon time transfer spread (PTS) to a minimum, below 10 ps. For the measurements with the 3 × 3 mm^2^ SiPMs we coupled the PbF_2_ crystal with Meltmount (refractive index of 1.582) to the SiPMs, similar to the measurements with BGO and LYSO:Ce, Mg. This ensures that the angular distribution of photons entering the SiPM is almost equal in both cases, and therefore, the SPTR obtained is a good estimate for the ‘real’ value with coupled scintillators. Measuring the 1 × 1 mm^2^ SiPMs we applied a small air-gap between crystal and detector.

**Figure 5.**
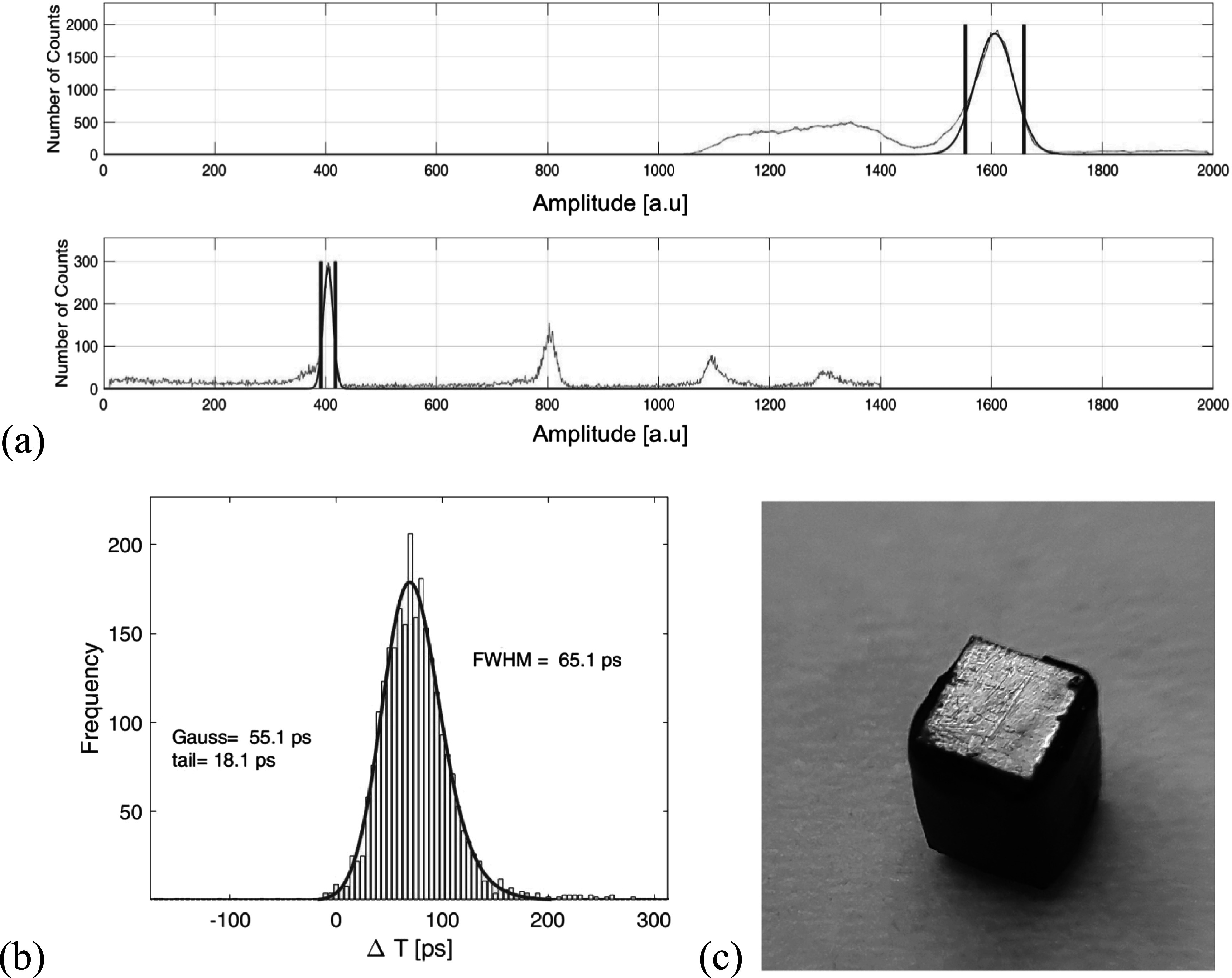
(a) Amplitude discrimination on the 511 keV photopeak for the reference channel and on single photoelectron signals on the NUV-HD-CHK SiPMs. (b) Obtained coincidence time spectra from which the CTR of the reference detector and electronic noise still has to be subtracted. (c) Picture of the black painted PbF_2_ crystal used for SPTR measurements.

In order to calibrate our setup, we measured two identical reference detectors of 2 × 2 × 3 mm^3^ LYSO:Ce,Mg coupled to NUV-HD-CHK LF2 and obtained a CTR of 66 ps FWHM. In the subsequent SPTR measurements we replaced one side with the SiPM under test and PbF_2_. In the data analysis we selected to photopeak events of the reference detector and to single photon avalanche diode signals (only one photon detected) of the SiPM under test. The according time delay histogram is then plotted and the SPTR fit modeled as a Gaussian convolved with an exponential tail, representing the diffusion tail of delayed carrier detection (see equation (1)) (Nemallapudi *et al* 2016). Representative plots are shown in figure 5.

\begin{eqnarray*}{\mathsf{SPTR}}(t)=\displaystyle \frac{1}{\sqrt{2\pi {\sigma }_{{\mathsf{SPTR}}}}}\cdot {e}^{-\tfrac{{\left(t-\mu \right)}^{2}}{2({\sigma }_{{\mathsf{SPTR}}}^{2})}}\ast \lambda {e}^{-\lambda t}.\end{eqnarray*}

The variable *μ* is assigned to the mean time delays within the electronics. Equation (1) can be represented via equation (2) (Nemallapudi *et al* 2016) where *λ* models the exponential characteristics of the delay tail and the error function is expressed in equation (3).

\begin{eqnarray*}{\mathsf{SPTR}}(t)=\displaystyle \frac{\lambda }{2}\cdot {e}^{\tfrac{\lambda }{2}(2\mu +\lambda {\sigma }_{{\mathsf{SPTR}}}^{2}-2t)}\cdot \left[1-{\mathsf{erf}}\left(\displaystyle \frac{\mu +\lambda {\sigma }_{{\mathsf{SPTR}}}^{2}-t}{\sqrt{2}{\sigma }_{{\mathsf{SPTR}}}}\right)\right]\end{eqnarray*}

\begin{eqnarray*}{\mathsf{erf}}(t)=\displaystyle \frac{2}{\sqrt{\pi }}{\int }_{0}^{t}{e}^{-{x}^{2}}{dx}.\end{eqnarray*}

From the measured SPTR in FWHM we deconvolve the time resolution of the reference detector (66 ps/$\sqrt{2}$ = 46 ps) and further the estimated electronic noise contribution (TR_SPAD_). To calculate TR_SPAD_ the noise floor (*σ*_noise_) of the analog signal and the slew rate (*dV*/*dt*) at a given leading edge threshold are determined, with ${\mathrm{TR}}_{\mathrm{SPAD}}=2.35\cdot {\sigma }_{\mathrm{noise}}/({dV}/{dt})$. It should be noted that the effect of the electronic noise is almost negligible for all measured configurations with the used HF-electronic readout.

### 2.5. Monte Carlo simulations

We compared the CTR measurements to published Monte-Carlo simulations, where a similar HF-readout concept and SiPMs were used (Gundacker *et al* 2020). The simulations take into account the light transport in the crystal, optical wrapping with Teflon and coupling with Meltmount (*n* = 1.582). Light ray tracing was done in SLitrani and Geant4, whereas the analog signal pile-up was implemented in a custom Matlab code, including the SPTR, PDE, DCR and optical crosstalk of the SiPM. The aim of the MC simulations is to compare a full analog readout (experimental setup in this work) to a hypothetical fully digital readout. The digital approach records all photons detected, sorts them in time and uses only the first photon detected as time-stamp estimation. The used optical simulations are exactly the same in both cases, which should give a fair comparison of analog and digital readout. Further information on the simulator can be found in Gundacker *et al* (2013, 2015), Acerbi and Gundacker (2019).

## 3. Results

### 3.1. Single photoelectron signals

**Figure 6.**
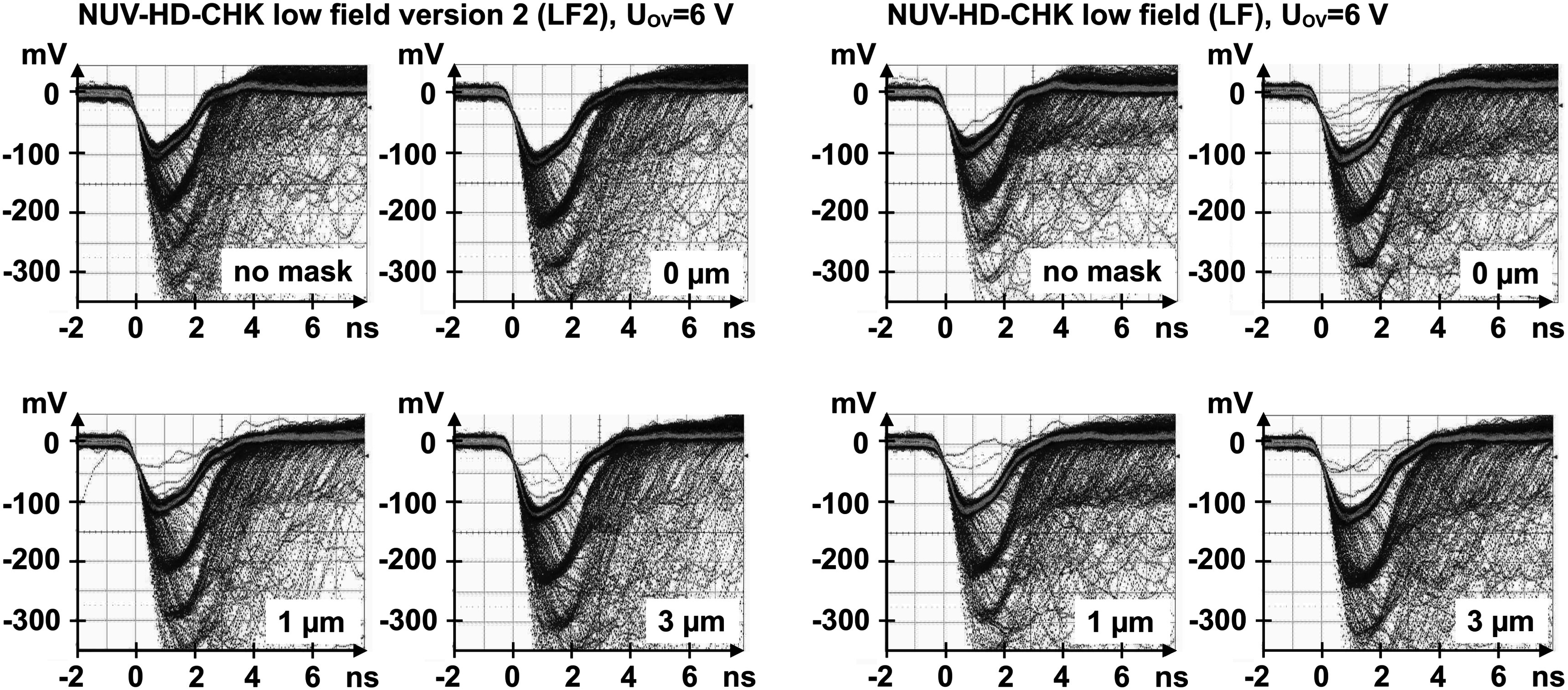
Comparison of the single-photoelectron signals obtained with different metal masking implemented on low field (LF) and low field version two (LF2) devices.

Each SiPM sample was tested without a crystal to obtain single-cell signals. Figure 6 shows single photoelectron signals obtained with different metal mask configurations, read out by the power efficient HF amplifiers. The metal mask increases the fast component of the micro-cell signals compared to the non-masked device, in both wafers W15 (LF) and W13 (LF2). Although there is a significant increase of the SPAD signals with increasing masking, the increase is in the 1%–10% range only. In the further discussions this increase of the single cell signals will not play a significant role in improving the CTR when coupling the scintillators.

### 3.2. Photon detection efficiency

**Figure 7.**
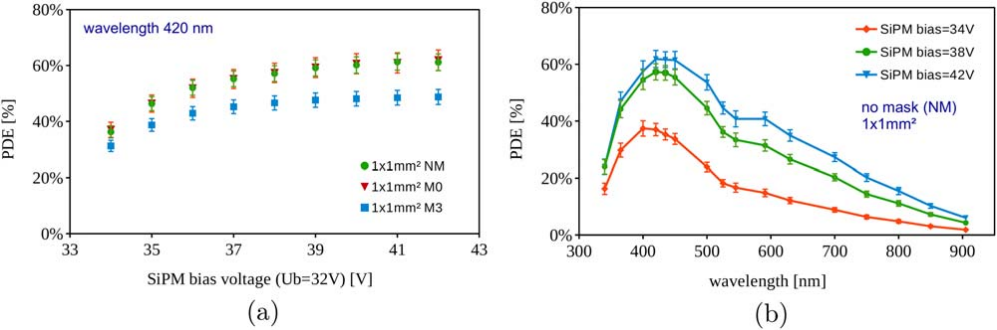
Measured PDE for the tested samples with different masking (NM, M0 and M3) of low field (LF) type. (a) PDE as a function of SiPM bias voltage at 420 nm and (b) PDE as a function of the wavelength.

To understand the effect of the masking on the photon detection we show in figure 7 the measured PDE as a function of SiPM bias voltage at 420 nm wavelength and as a function of wavelength at three selected bias voltages. Devices used for the measurements where 1 × 1 mm^2^ sized of low field (LF) type. The plots show that NM (no masking) and M0 (0 *μ*m mask overlap) show exactly the same PDE with maximum values around 60% at 38 V, whereas M3 (3 *μ*m mask overlap) shows a PDE of about 46%. In comparison, from pure geometric considerations, we can calculate that M1 and M3 will have a relative PDE loss of 89.4% and 70.3%, respectively.

### 3.3. Energy resolution performance

For each coincidence event, digitized energy signals were integrated and the resulting energy values filled in a histogram to form the energy spectrum. An energy resolution at 511 keV for each SiPM sample was calculated and compared to those of other SiPM samples. BGO coupled to SiPMs with 3 *μ*m masking showed slightly better energy resolution than the others. The energy resolution measured ranged from 17% to 19% FWHM.

### 3.4. CTR with pole-zero operational amplifier setup

**Figure 8.**
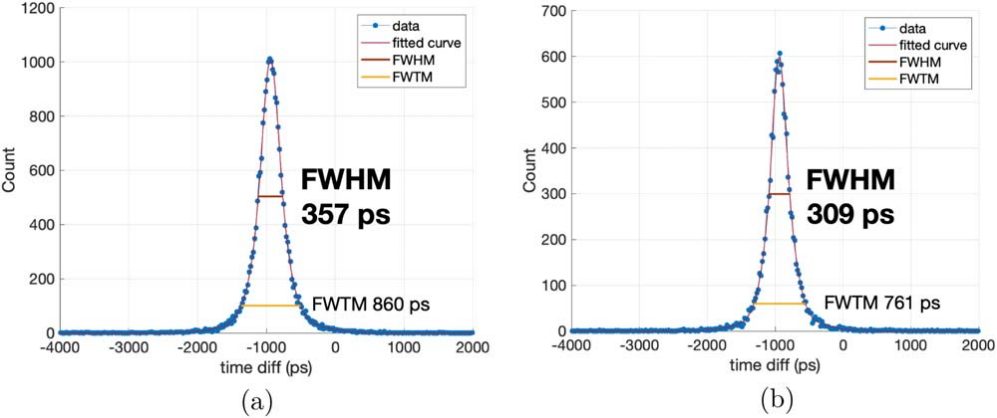
Timing spectra measured with the operational trans-impedance amplifier setup, using SiPMs with no mask from W15 (a) and with 0 *μ*m masking from W13 (b) coupled to 3 × 3 × 5 mm^3^ BGOs.

Better CTRs were obtained with the masked SiPMs. Based on the CTR results measured with the reference detector, we selected two SiPM with no-mask from W15 (low field) and two SiPMs with 0 *μ*m masking from W13 (low field v2). Figure 8 shows timing spectra obtained from two different SiPM pairs. When measuring with an operational amplifier based front-end circuit and with 3 × 3 × 5 mm^3^ the CTR of 0 *μ*m masked SiPMs from W13 coupled to BGO was evaluated to be better than that of no-mask SiPMs. The 3 *μ*m masked SiPM results are comparable to no-mask measurements, most likely due to an additional light loss caused by the thicker masking.

### 3.5. CTR with HF-readout

An overview of the measured CTR using HF-electronics with the different NUV-HD-CHK SiPMs can be seen in table 1. The CTR was evaluated with BGO from EPIC-crystals and with LYSO:Ce,Mg from Taiwan applied crystals (TAC) of 2 × 2 × 3 mm^3^ and 3 × 3 × 20 mm^3^ size. In table 1 we further compare the values obtained with the novel NUV-HD-CHK SiPMs to FBK’s standard field (SF) devices, without masking of 4 × 4 mm^2^ active area and 40 *μ*m SPAD pitch, and to commercially available devices from Broadcom (AFBR-S4N33C013) of 3 × 3 mm^2^ active area and 30 *μ*m SPAD pitch, as well as to HPK S14160-3050HS of 3 × 3 mm^2^ active area and 50 *μ*m SPAD pitch.

**Table 1.** Overview of the CTRs measured with BGO and LYSO:Ce,Mg of different sizes obtained with the different SiPMs tested at 38 V bias (6 V overvoltage). Breakdown of the SiPMs are 32 V for FBK NUV-HD-CHK, 28 V for FBK NUV-HD, 26.5 V for Broadcom and 38 V for HPK. The statistical error is around ±3 ps FWHM.

SiPM	bias	CTR [ps]	CTR [ps]	CTR [ps]	CTR [ps]	relative
	[V]	LYSO:Ce, Mg	BGO	LYSO:Ce, Mg	BGO	PDE
		2 × 2 × 3 mm^3^	2 × 2 × 3 mm^3^	3 × 3 × 20 mm^3^	3 × 3 × 20 mm^3^	[%]
NUV-HD-CHK LF2	38	66	126	107	256	100
NUV-HD-CHK LF2 M0	38	63	128	106	245	100
NUV-HD-CHK LF2 M1	38	64	123	108	243	89.4
NUV-HD-CHK LF2 M3	38	64	130	115	265	70.3

NUV-HD SF	37	69	136	113	265	100
Broadcom	37	75	148	119	273	-
(AFBR-S4N33C013)						
Hamamatsu—HPK	46	-	159	-	326	-
(S14160-3050HS)						

The best performance was achieved with the low field version 2 (LF2) devices and 1 *μ*m masking. The CTR in FWHM with the short 3 mm long BGO is 123 ps, whereas an increased length to 20 mm still shows a CTR of 243 ps FWHM. It can be seen that a larger mask of 3 *μ*m leads to worse CTR values most likely due to the larger loss in relative PDE, as can be seen in table 1. The specially designed NUV-HD-CHK devices perform better than commercially available SiPMs from Broadcom as well as HPK and further outperform the standard field FBK NUV-HD devices.

**Figure 9.**
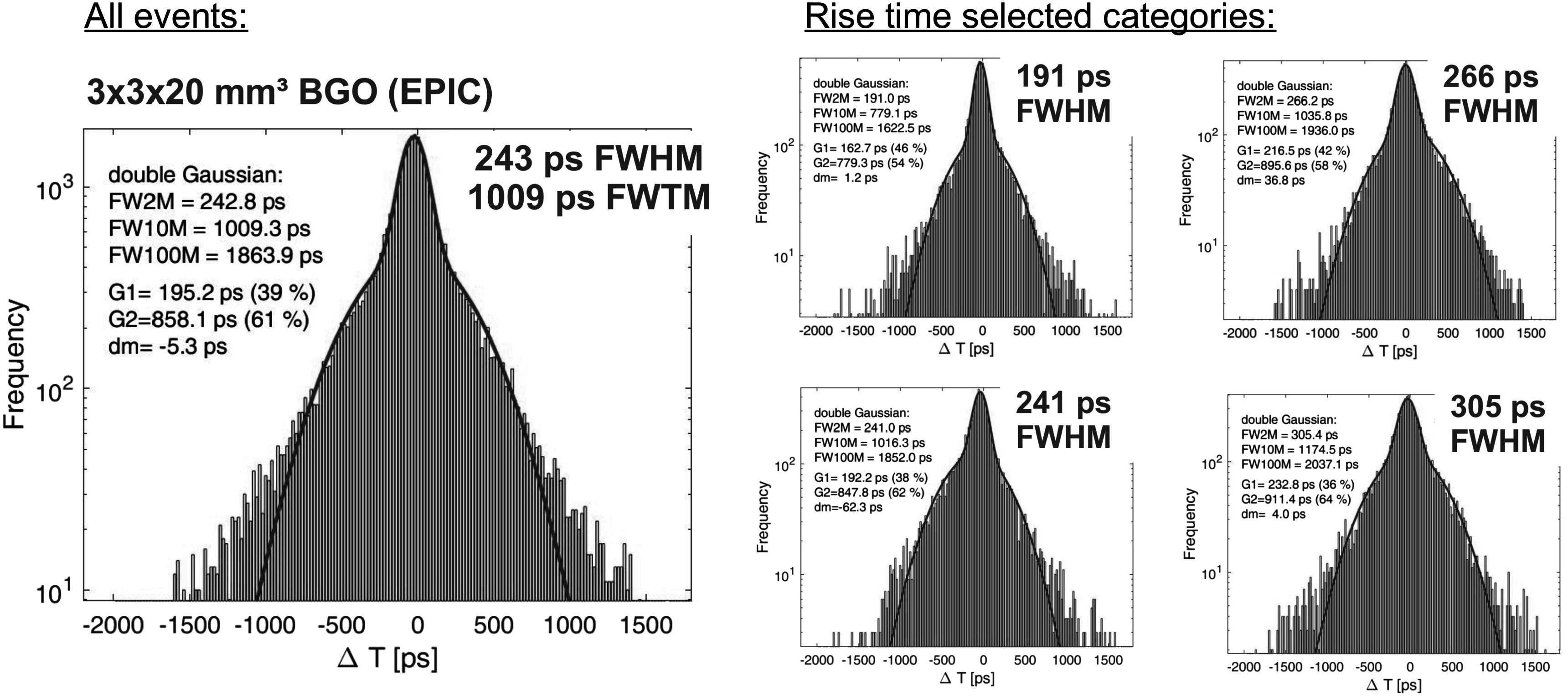
Coincidence time resolution measured with NUV-HD-CHK and 1 *μ*m masking coupled to BGO of 3 × 3 × 20 mm^3^ size wrapped in Teflon. A CTR of 243 ps FWHM can be achieved in this configuration. If selecting to the rise times of the timing signal, the best 25% of coincident events show a CTR of 191 ps FWHM.

**Figure 10.**
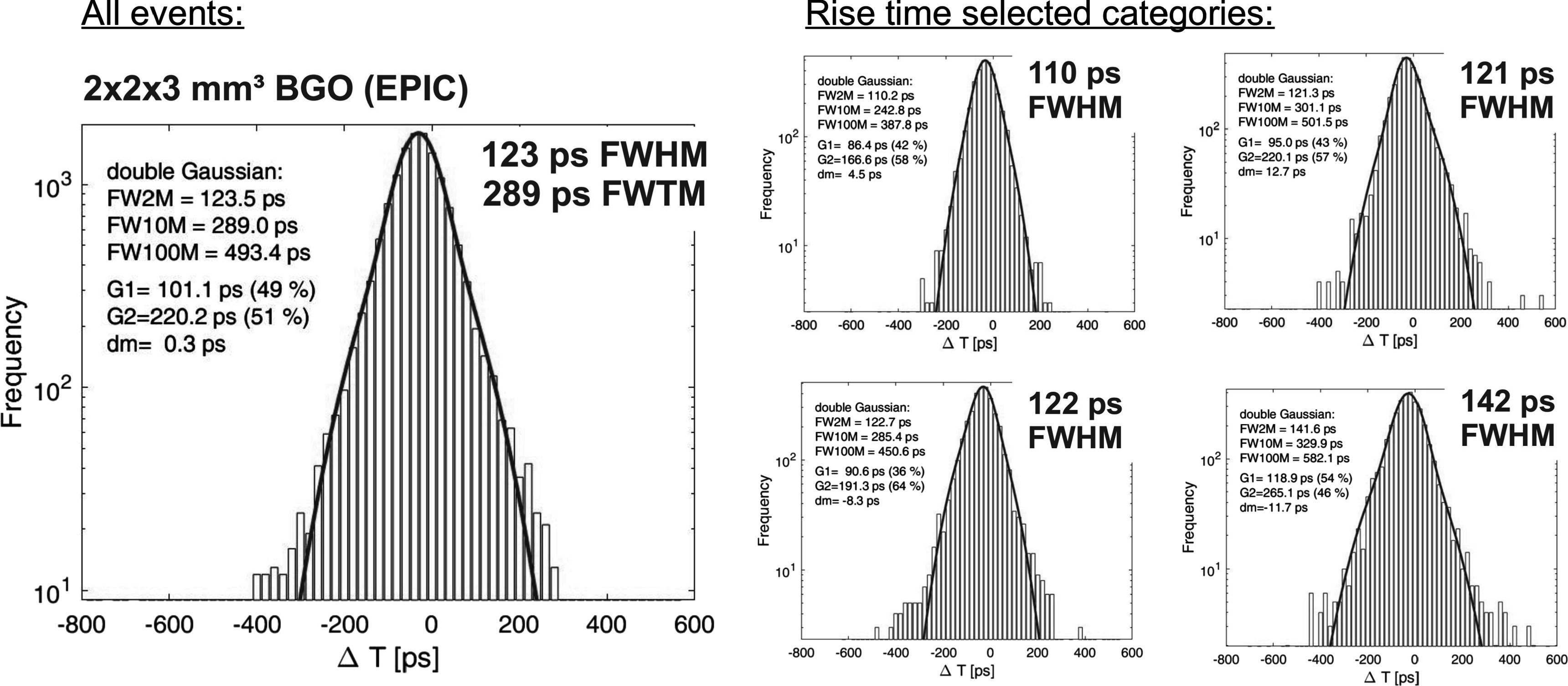
Coincidence time resolution measured with NUV-HD-CHK and 1 *μ*m masking coupled to BGO of 2 × 2 × 3 mm^3^ size wrapped in Teflon. A CTR of 123 ps FWHM can be achieved in this configuration. If selecting to the rise times of the timing signal, the best 25% of coincident events show a CTR of 110 ps FWHM.

In order to illustrate the effect of the time walk classification we show in figure 9 the CTR histogram with BGO of 3 × 3 × 20 mm^3^ size coupled to NUV-HD-CHK (LF2) of 1 *μ*m masking. On the left the histogram shows all events combined, whereas on the right hand side the CTR is divided into four categories, selected by the signal rise time, as described in Kratochwil *et al* (2020). In this way we obtain for the best 25% of coincidence events a CTR of 191 ps FWHM, whereas the slowest 25% of events show a CTR of 305 ps FWHM. A similar behavior is shown in figure 10 for 2 × 2 × 3 mm^3^ crystal size, where the nominal CTR is 123 ps FWHM and the best 25% of events achieve 110 ps FWHM. It should be stressed that by this method no events were discarded, hence, the sensitivity in a PET system would remain unchanged.

### 3.6. SPTR with HF

An overview of the measured SPTRs can be seen in table 2. Besides the measured SPTR the intrinsic SPTR is stated, for which the electronic noise component of the HF-electronics is subtracted following the method outlined in Gundacker *et al* (2020). The electronic noise floor was 4 mV rms and a typical slew rate is 0.3 mV ps^−1^ or 300 V *μ*s^−1^ for single SPAD signals at optimum bias (38 V), which gives an electronic noise contribution (TR_SPAD_) of around 30 ps FWHM to the SPTR for these large 3 × 3 mm^2^ devices.

**Table 2.** Overview of the best SPTR measured with black painted PbF_2_ and the intrinsic SPTR, which is the measured SPTR corrected for electronic noise. The relative PDE, calculated from the geometric fill factor is given as well. Breakdown of the SiPMs are 32 V for FBK NUV-HD-CHK, 28 V for FBK NUV-HD, 26.5 V for Broadcom and 38 V for HPK. Statistical error-bars are within ±2 ps.

SiPM	bias	SPTR [ps]	SPTR [ps]	relative
	[V]	measured with	without	PDE
		PbF_2_	elec. noise	[%]
NUV-HD-CHK LF2	38	73.0	65.1	100
NUV-HD-CHK LF2 M0	38	60.7	49.9	100
NUV-HD-CHK LF2 M1	38	58.5	46.7	89.4
NUV-HD-CHK LF2 M3	38	52.3	41.9	70.3

NUV-HD-CHK LF	38	74.4	64.5	100
NUV-HD-CHK LF M0	38	65.7	56.4	100
NUV-HD-CHK LF M1	38	62.9	51.5	89.4
NUV-HD-CHK LF M3	38	53.8	42.0	70.3

NUV-HD SF	38	72.7	68.5	100
Broadcom AFBR-S4N33C013	38	69.5	65.3	-
HPK S14160-3050HS	46	127.4	125.9	-

In table 2 a clear correlation of the SPTR with the level of masking can be noticed, with the best values achieved by masking 3 *μ*m, showing an intrinsic SPTR of 42 ps FWHM. Intrinsic SPTR values obtained for the standard field FBK device of 68 ps, Broadcom SiPM of 65 ps and HPK SiPM of 126 ps are similar to values obtained in literature, measured with a picosecond laser (Gundacker *et al* 2020). This shows that the proposed method of measuring the SPTR with a black painted PbF_2_ crystal is a valid approach, and even more precise due to the exact knowledge of the reference detector’s time resolution. Furthermore, it can be seen that the low field v2 (LF2) devices show slightly better SPTR, however, the difference lies almost within the statistical errors.

**Figure 11.**
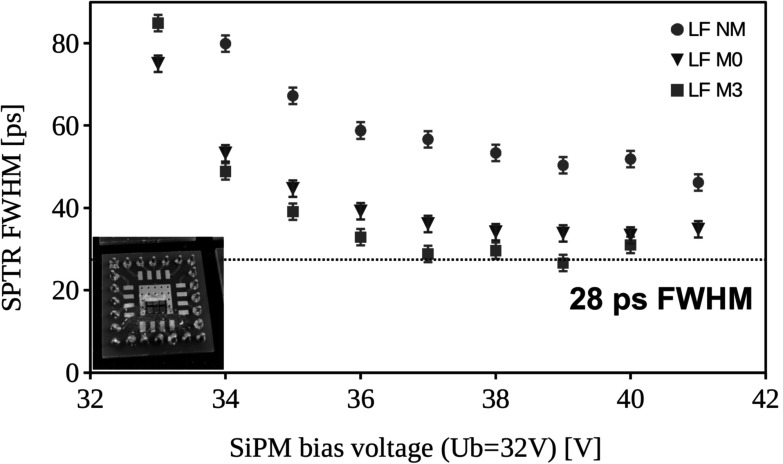
Single photon time resolution measured with 1 × 1 mm^2^ low field NUV-HD-CHK devices and different masking (no mask—NM, 0 *μ*m - M0 and 3 *μ*m mask—M3). The plotted SPTR values are not corrected for the electronic noise contribution, which however is negligible due to the small SiPM terminal capacitance and large SPAD signals.

Achieving SPTR values of 42 ps FWHM with 3 × 3 mm^2^ sized SiPMs further opens the question on how much these values are influenced by electronic signal transfer time spreads, i.e. the size of the SiPM. In order to get an idea, we tested smaller SiPMs of 1 × 1 mm^2^ of the low field (LF) type and different masking, with SPTR results shown in figure 11. The best SPTR achieved is 28 ps FWHM with 3 *μ*m masking, whereas the 0 *μ*m masked SiPM does not perform much worse. On the other hand, the SiPM without masking (NM) shows much deteriorated SPTR values around 50 ps FWHM. It should be noted that these values are not corrected for the electronic noise contribution, which however, is negligible due to the large single photon signals of the small SiPMs (low terminal capacitance).

## 4. Discussion

**Figure 12.**
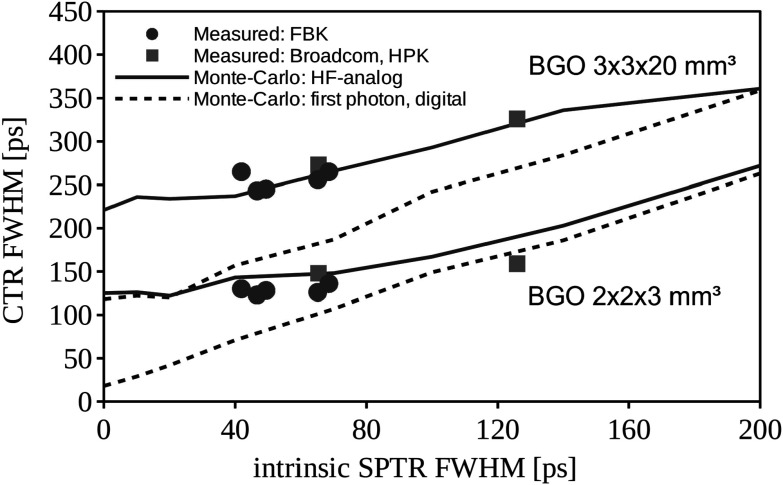
Monte-Carlo simulation of the CTR with BGO as a function of SPTR for the analog SiPM with leading-edge time estimation, shown along with the digital SiPM using only the first photon detected for the time estimation. Monte-Carlo simulation values are taken from Gundacker *et al* (2020). SPTR and CTR measurements have been performed in this work with a power-efficient HF-readout (tables 1 and 2).

To summarize our obtained results with BGO, we plot in figure 12 comprehensive Monte-Carlo simulations of the CTR achievable with BGO of 2 × 2 × 3 mm^3^ and 2 × 2 × 20 mm^3^ size with the SPTR varying from 0 to 200 ps FWHM. For the digital SiPM simulations in figure 12 we only use the first photon detected to estimate the 511 keV gamma emission time. The green circles and blue squares represent new measurements performed in this work, summarized in tables 1 and 2. It can be seen that the HF-analog simulations predict well the CTRs obtained by our experiments, which gives confidence that also our digital-SiPM simulations are properly implemented. We further realize that there is still plenty of room for improvements with the measured CTR values being dominated by the analog readout strategy of the SiPM signal and less by the SPTR. This also explains the rather marginal improvement of the CTR obtained with BGO from 126 to 123 ps for 2 × 2 × 3 mm^3^ and 256 to 243 ps for 3 × 3 × 20 mm^3^, with the FBK NUV-HD-CHK LF2 no mask and 1 *μ*m mask, respectively, as compared to the large SPTR improvement from 65 to 46 ps FWHM with masking. As can be seen in figure 12 an SPTR of 46 ps FWHM would allow for a CTR of 150 ps for 20 mm and 60 ps for 3 mm long BGO scintillators in the case of digital readout (or negligible analog readout contribution), which would be well below the state-of-the-art with LYSO:Ce in systems (Conti and Bendriem 2019).

Increasing the masking to 3 *μ*m (FBK LF2 M3) even shows a deteriorated CTR in table 1 and figure 12 despite the best obtained intrinsic SPTR value of 42 ps FWHM (table 2). This behavior can be concisely explained by the lowest fill factor and hence relative PDE of 70% for this device. In conclusion, a refined balance between SPTR and PDE has to be found in the design of the masking.

In figure 8 the CTR achieved with an operational amplifier setup was shown, based on a standard inverting amplifier configuration. The bandwidth of this circuit is limited to about 300–500 MHz and has similarities to a current-mirror readout of the SiPM signal. Hence, this circuit can be seen as a working model for available ASIC solutions with similar bandwidths and current-mirror implementation. Comparing the timing results with this circuit for 5 mm long crystals to the HF-readout, it is obvious that the HF-readout brings an enormous advantage in terms of best achievable CTR, with even better values achieved for 20 mm length. This advantage is caused by the higher bandwidth in the HF-readout and further by a faster slew rate of the signal, at the cost of a high power consumption.

Whereas the LYSO:Ce measurements show a pure Gaussian shape in the CTR histogram, the ratio FWHM over FWTM is on the other hand an important parameter for BGO and its use in imaging systems. This ratio is similar for short crystal for the operational amplifier readout with values of 0.42 (5 mm length) and for the HF-readout with 0.43 (3 mm length), to be see in figure 8 and 10, respectively. Increasing the crystal length to 20 mm decreases the FWHM/FWTM ratio to 0.24, as can be seen in figure 9. Most likely this is caused by the lower light transfer efficiency (LTE) in longer crystals, resulting in a higher probability of zero Cherenkov photons being detected, which causes the rather slow BGO scintillation to be more prominent. This can also be seen in an abundance decrease of the fast Gaussian with long crystals, as compared to short crystals.

Regarding the highest achievable SPTR with masked SiPMs, we showed in figure 11 that smaller devices of 1 × 1 mm^2^ size can achieve values of 28 ps FWHM as compared to 42 ps FWHM for 3 × 3 mm^2^ SiPMs (table 2). From these tests two important conclusions can be drawn: (i) the electronic signal transfer plays a crucial role in achieving highest SPTR in large area SiPMs, especially when the aim is to reach values below 20 ps FWHM, (ii) masking helps in suppressing this signal transfer time spread (huge SPTR improvement by masking compared to no mask), which indeed seems to be the governing contribution with the SPAD edges playing an inferior role (almost same SPTR for masking with 0 *μ*m and 3 *μ*m). Considering this experimental results we conclude that masking is primarily important for a fast extraction of the signal, especially in large area SiPMs, but should play an inferior role when smaller SiPM areas are to be considered. However, this assumption only is proven for SPTR values larger 20 ps. If values below that should be achieved it is thinkable that the SPAD edges start to play a major contribution on the SPTR. Nevertheless, achieving sub-30 ps SPTR seems to be possible with small area SiPMs regardless of the masking overlap, which allows for higher PDE and therefore better CTR.

## 5. Conclusions

In this work we introduced developments to improve the well established NUV-HD technology from FBK for Cherenkov detection, with focus on BGO. The SPAD field configuration was optimized and the SiPM surface covered with a metal mask to improve the SPTR on the individual SPAD level, by shielding their edges, and on the SiPM level to allow for a fast signal transfer to the terminal connections. Furthermore, the metal mask effectively increased the fast component of the single-cell signal, resulting in improved signal slew-rates, although this effect is of secondary order. Different types of masking were tested and the SPTR was found to correlate with the masking size and best values of 42 ps FWHM were obtained for a 3 *μ*m overlap with the SPAD active area and 3 × 3 mm^2^ device area. Measuring with BGO and LYSO:Ce, Mg crystals a trade-off between SPTR and PDE has to be maintained for which a smaller overlap of 1 *μ*m into the SPAD’s active area gave best performances. The newly developed SiPMs of 3 × 3 mm^2^ size allowed to improve the CTR with BGO to 243 ps FWHM for 3 × 3 × 20 mm^3^ crystals, as compared to standard devices with 265 ps. CTR values with 3 × 3 × 20 mm^3^ LYSO:Ce, Mg crystals reached 106 ps FWHM. It was further shown that smaller SiPMs of 1 × 1 mm^2^ and 3 *μ*m masking can reach SPTRs of 28 ps FWHM. These unprecedented values on SiPM level opens the door to superb timing with prompt Cherenkov photons, which will be subject for future studies and applications.

## Data Availability

All data that support the findings of this study are included within the article (and any supplementary information files). Data will be available from 13 February 2024.
